# Biologically Compatible Lead-Free Piezoelectric Composite for Acoustophoresis Based Particle Manipulation Techniques

**DOI:** 10.3390/s21020483

**Published:** 2021-01-12

**Authors:** Tomas Janusas, Sigita Urbaite, Arvydas Palevicius, Sohrab Nasiri, Giedrius Janusas

**Affiliations:** Faculty of Mechanical Engineering and Design, Kaunas University of Technology, Studentu str. 56, LT–51424 Kaunas, Lithuania; tomas.janusas@ktu.lt (T.J.); sigita.urbaite@ktu.lt (S.U.); arvydas.palevicius@ktu.lt (A.P.); sohrab.nasiri@ktu.lt (S.N.)

**Keywords:** lead-free, BCZT, microchannel, bulk acoustic waves, particle manipulation

## Abstract

This research paper is concentrated on the design of biologically compatible lead-free piezoelectric composites which may eventually replace traditional lead zirconium titanate (PZT) in micromechanical fluidics, the predominantly used ferroelectric material today. Thus, a lead-free barium–calcium zirconate titanate (BCZT) composite was synthesized, its crystalline structure and size, surface morphology, chemical, and piezoelectric properties were analyzed, together with the investigations done in variation of composite thin film thickness and its effect on the element properties. Four elements with different thicknesses of BCZT layers were fabricated and investigated in order to design a functional acoustophoresis micromechanical fluidic element, based on bulk acoustic generation for particle control technologies. Main methods used in this research were as follows: FTIR and XRD for evaluation of chemical and phase composition; SEM—for surface morphology; wettability measurements were used for surface free energy evaluation; a laser triangular sensing system—for evaluation of piezoelectric properties. XRD results allowed calculating the average crystallite size, which was 65.68 Å^3^ confirming the formation of BCZT nanoparticles. SEM micrographs results showed that BCZT thin films have some porosities on the surface with grain size ranging from 0.2 to 7.2 µm. Measurements of wettability showed that thin film surfaces are partially wetting and hydrophilic, with high degree of wettability and strong solid/liquid interactions for liquids. The critical surface tension was calculated in the range from 20.05 to 27.20 mN/m. Finally, investigations of piezoelectric properties showed significant results of lead-free piezoelectric composite, i.e., under 5 N force impulse thin films generated from 76 mV up to 782 mV voltages. Moreover, an experimental analysis showed that a designed lead-free BCZT element creates bulk acoustic waves and allows manipulating bio particles in this fluidic system.

## 1. Introduction

Many applications are looking for the ability to move a particle or a fluid mixture into some separate components in microfluidic devices. This might be reached by implementing certain exclusion filters letting the fluid to flow and trapping the particles on it [1,2,3]. However, there are certain limitations when using such filters: reduced filtration capacity when the filter is filled up, changing or removing the filter, fixing trapped particles in filters, etc. [4]. The main challenge of these devices is related to the design of effective fluid manipulation techniques having a large variety of biomolecules (e.g., bacteria, cells, etc., vary in size from ≈1 to 30 µm) and different suspending mediums (blood, cells, sputum, etc.). Recent research has shown, that there are two main particle manipulation techniques widely discussed—active (e.g., acoustophoresis) [3,5] and passive (e.g., internal microfluidics) [6,7], both of them are used in diagnostic and medical applications. Passive techniques are based on hydrodynamic particle manipulation by tuning geometry and fluid; active ones—by acoustic manipulation. Both are widely applied, discussed, and examined; however, they have certain limitations such as poor separation efficiency for particle-rich analytes or fluids, and particle size (typical range from 1 to 20 µm), thermal stresses in material during the flow process which may destructs particles, etc.

The aim of this research paper is to propose a technology based on the principle of bulk acoustic wave generation. It is related to the development of lead-free piezoceramics [8,9,10] which may be applied in the process of acoustophoresis [3,4,5]. Acoustophoresis is a noncontact and label-free mode of manipulation process separating the particles using high intensity sound waves and eliminating the necessity of earlier mentioned exclusion filters or membranes in microfluidic devices. Acoustic manipulation exerts forces on particles when high intensity standing waves act. This standing wave stands still in time with a pressure profile at its nodes and antinodes containing areas of net zero pressure [11]. Thus, moving particles, depending on their size and density, may be trapped at these nodes. Therefore, traditional acoustophoresis devices must be improved. Development of lead-free piezoceramics by replacing traditional lead zirconium titanate (PZT) ceramics [12,13,14] has gained huge interest in the field of biologically compatible manipulation techniques.

PZT for over 50 years has been a very popular and widely studied ceramic allowing to synthesize a large number of materials with different properties due to the range of Zr:Ti ratios used in synthesis process [12,13,14,15]. However, in the past few years the toxicity of lead oxide was highly discussed encouraging the development of lead-free piezoceramics and devices for medical, pharmaceutical, and other applications. There are known prospective lead-free material systems as (Bi_1/2_K_1/2_)TiO_3_ (BKT) [16], (Bi_1/2_Na_1/2_)TiO_3_ (BNT) [16], sodium potassium niobate (KNN) [17], etc., showing piezoelectric constant d_33_ of 416 pC/N similar to that of PZT. However, the main problem appears during calcinations and sintering processes when some components (as, e.g., the alkali in KNN system) become highly volatile at even very small temperatures. It gets difficult to maintain the same processes because of chemical stoichiometry problems. Thus, no effective alternatives to PZT were designed, yet, making this topic of high importance among researchers.

In this research paper, lead-free barium–calcium zirconate titanate Ba_0.85_Ca_0.15_(Zr_0.1_Ti_0.9_)O_3_ (BCZT) piezoelectric ceramic was designed and engineered. The dependency of thin film thickness to design element properties was investigated too. A modified oxalate–hydroxide coprecipitation method was used to synthesize barium–calcium zirconate titanate nanocrystalline powder. Four samples with different thicknesses of BCZT thin composite layers (named as Ba32, Ba48, Ba90, and Ba140) were fabricated and investigated in order to design a functional acoustophoresis micromechanical fluidic element, based on the principle of bulk acoustic generation for particle control technologies. Based on the scientific papers [18,19], materials used for synthesis are biologically compatible. Fourier transform infrared (FTIR) and X-ray diffraction (XRD) techniques were used to evaluate a chemical-phase composition of BCZT composite; scanning electron microscopy (SEM) was used for surface morphology investigations; wettability measurements were used to find its surface free energy (SFE); a laser triangular sensing system was used for evaluation of piezoelectric properties. Further, experimental research in the design of a microchannel for particle motion manipulation using bulk acoustic waves was undertaken. A lead-free piezoceramic was implemented in an acoustic manipulation system, proving its suitability for such applications as continuous flow microsystems, medical diagnostics, analysis, etc. Designed lead-free BCZT based microchannel creates multidimensional bulk acoustic waves and propels the particles towards the channel allowing to control the migration and concentration of particles’ kinetics. This feature is desirable in such microsystem devices where scalability, programmability, and the ability to control variable size particles is crucial.

## 2. Materials and Methods

### 2.1. Synthesis of Lead-Free BCZT

A modified oxalate–hydroxide coprecipitation method was used for synthesis of barium–calcium zirconate titanate nanocrystalline powder. Materials used were as follows: barium acetate Ba(CH_3_COO)_2,_ calcium acetate monohydrate Ca(CH_3_COO)_2_ H_2_O, tetra-n-butyl-titanate Ti (OC_4_H_9_)_4,_, tetra-n-butyl zirconate Zr(OC_4_H_9_)_4_ (80% solution in n-butanol), oxalic acid dihydrate H_2_C_2_O_4_, H_2_O, and deionized water.

Thus, 14.2 g of barium acetate and 4.7 g of calcium acetate monohydrate were dissolved in 100 mL of deionized water at room temperature. A total of 49.0 g (slight excess) of oxalic acid dihydrate were dissolved in 500 mL of deionized water in a 1-L beaker and solution was warmed up to 50 °C. In total, 38.2 g of tetra-n-butyl-titanate and 3.9 g of tetra-n-butyl-zirconate were added dropwise into oxalic acid solution and stirred vigorously until clear yellow solution was obtained. Both Zr and Ti butylates react with oxalic acid according to Equations (1) and (2) to give soluble intermediate products of reaction:

(1) $${\mathrm{Zr}(\mathrm{OC}}_{4}H_{9}{)}_{4}{\;+\;2\;H}_{2}C_{2}O_{4}{\;+\;H}_{2}O\;\to {\;H}_{2}{\mathrm{ZrO}(C}_{2}O_{4}{)}_{2}{\;+\;4\;C}_{4}H_{9}\mathrm{OH};$$

(2) $${\mathrm{Ti}(\mathrm{OC}}_{4}H_{9}{)}_{4}{\;+\;2\;H}_{2}C_{2}O_{4}{\;+\;H}_{2}O\;\to H_{2}{\mathrm{TiO}(C}_{2}O_{4}{)}_{2}{\;+\;4\;C}_{4}H_{9}\mathrm{OH};$$

After stirring for 1 h, combined solution of barium and calcium acetates was poured into this solution and white precipitate of insoluble calcium and barium oxalates was formed according to Equations (3) and (4):

(3) $${\mathrm{Ca}(\mathrm{CH}}_{3}{\mathrm{COO})}_{2}{\;+\;H}_{2}C_{2}O_{4}\to {\mathrm{CaC}}_{2}O_{4}{\;+\;2\;\mathrm{CH}}_{3}\mathrm{COOH};$$

(4) $${\mathrm{Ba}(\mathrm{CH}}_{3}{\mathrm{COO})}_{2}{\;+\;H}_{2}C_{2}O_{4}\;\to {\mathrm{BaC}}_{2}O_{4}{\;+\;2\;\mathrm{CH}}_{3}\mathrm{COOH};$$

In the next stage, the soluble titanyl- and zirconyl oxalates reacts with freshly formed oxalates of barium and calcium forming white insoluble precipitate of mixed calcium(barium) zirconyl(titanyl) oxalates:

(5) $${\mathrm{Ca}(\mathrm{Ba})C}_{2}H_{4}{\;+\;H}_{2}{\mathrm{ZrO}(\mathrm{TiO})(C}_{2}H_{4}{)}_{2}\;\to {\mathrm{Ca}(\mathrm{Ba})\mathrm{ZrO}(\mathrm{TiO})(C}_{2}H_{4}{)}_{2}{\;+\;H}_{2}C_{2}O_{4};$$

After that, reaction mixture was stirred for another 2 h at room temperature. In contrast to synthesis of PZT done in previous research [12,13], 25% ammonia solution was not added to reaction mixture at this stage with purpose to avoid formation of quite soluble hydroxides of barium (4 g/100 mL at 20 °C) and calcium (1.73 g/L at 20 °C). The white amorphous powder was filtered under vacuum, washed thoroughly with deionized water and acetone, and dried at 100 °C for 12 h. The dry powder was calcinated at 1000 °C for 10 h. The white nanocrystalline powder of barium–calcium zirconate titanate was obtained with 97% (9.7 g).

### 2.2. Formation of BCZT Elements

**Figure 1:**
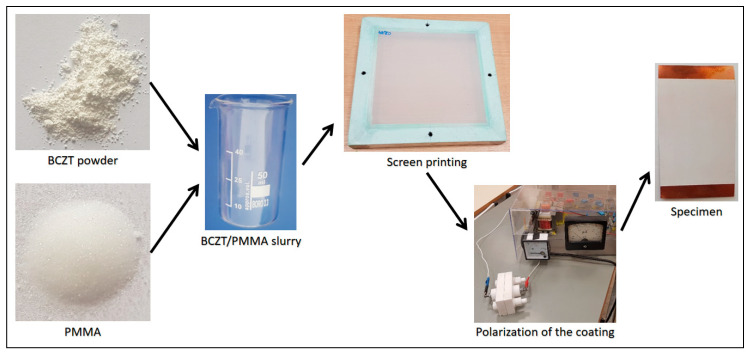
General fabrication scheme for formation of BCZT based elements.

A general scheme for formation of BCZT elements with different BCZT thin film layer thicknesses is presented in Figure 1.

**Figure 2:**
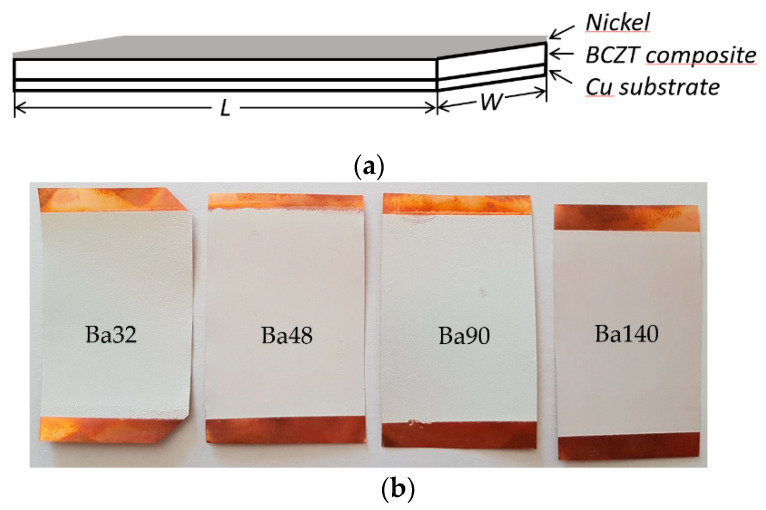
Scheme of (**a**) sandwich panel of BCZT composite based on element and (**b**) four fabricated samples using different screen printing meshes.

The first step in fabrication of BCZT elements was preparation of BCZT/PMMA slurry mixture in ratio 80:20, respectively. The ratio was chosen based on previous research related to the poly(methyl methacrylate) (PMMA) and its synthesis with other piezoceramic materials [20]. Further, thin film layers were formed via screen printing technique with four different mesh frames on copper (Cu) plates of 50 µm thicknesses and poled at an electric field of 10 MV m^−1^. A copper plate here acts as a bottom electrode, thus a nickel thin film layer of 10 nm formed on the BCZT layer acts as a top electrode (see Figure 2).

Experimental results implied that using different meshes in screen printing technology it is possible to form smooth thin film layers of BCZT composite on the substrate. Thus, four elements, Ba32 with measured BCZT layer thickness 109 ± 5 µm, Ba48 with thickness of 102 ± 5 µm, Ba90 with thickness 86 ± 5 µm, and Ba140 with thickness of 70 ± 5 µm, were designed (see Table 1).

### 2.3. Formation of a Microchannel

Lead-free BCZT/PMMA slurry mixture in ratio 80:20 (described in previous section) was used for formation of a microchannel. A thin film layer of 200 µm thicknesses was fabricated using screen printing technique with mesh No. 32/83–70 PW on a copper (Cu) plate of 50 µm thickness. A microchannel was formed using thermal imprint technique: embossing temperature was 150 °C, pressure force 5000 N, and embossing time was 10 s. Additionally, sonotrode and high frequency oscillations were used to ensure the quality of microchannel. Further, the formed microchannel was poled at an electric field of 10 MV m^−1^ and metalized with nickel film of 10 nm thickness (T_melting_ = 1455 °C) using electron beam evaporation technique. A copper plate here acts as a bottom electrode, while a nickel thin film layer acts as a top electrode Cover glass with inlet and outlet connections is glued on the top of microchannel. The whole system was glued to a glass plate, which ensures the stability of the system. This glass base ensures that bending and twisting vibrations are eliminated and allows assessing whether the particles are actually controlled by bulk acoustic waves.

### 2.4. Methods and Techniques for Properties Evaluation

XRD technique. The phase composition and purity of the materials were determined by X-ray diffraction spectrometer. The XRD patterns were investigated through the X’Pert software.

The lattice constant *a* can be calculated using the equation [9]:

(6) $$a=d\sqrt{h^{2}+k^{2}+l^{2}};$$

where, *h*, *k*, and *l* are indexes of the XRD reflection peak, and *d* is the interplaner spacing.

Further, calculations of the average BT crystallite diameter *D_XRD_* can be estimated from the equation [9] below, using the data of most intense (110) peak.

(7) $$D_{XRD}=\frac{c\lambda }{B_{1/2}\cos\theta };$$

where, *C*—constant; *B*_1/2_—the full width of the half maximum in 2*θ*; *θ*—corresponding Bragg angle.

FTIR for chemical composition analysis. Fourier Transform Infrared Spectrometer (Spectrum GX 2000 Raman) was used for characterization of molecules in chemical compounds. The diapason of FTIR spectra region was from—400 to 4000 cm^−1^ with a resolution of −1 cm^−1^.

SEM for energy distribution and surface morphology analysis. With a help of scanning electron microscope (Quanta 200 FEG) equipped with the Energy Dispersive X-ray Spectrometer (XFlash 4030 from Bruker, Billerica, MA, USA), morphological structure and chemical composition of a lead-free piezoelectric material was evaluated. Investigations were performed under controlled pressure water steam atmosphere. Maximal achievable resolution for high-vacuum (<6 × 10^−4^ Pa) is 0.8 nm, for low-vacuum (10 to 130 Pa)–1.5 nm, and for extended vacuum mode (10–4000 Pa)–1.5 nm. A modern 30 mm^2^ area solid state drift detector was cooled with Peltier element and provided 133 eV (at Mn K) energy resolution at 100,000 cps. Using X-ray spectroscopy, energy differences were measured between the various quantum states of a system together with the probabilities that the system jumps between these states.

**Figure 3:**
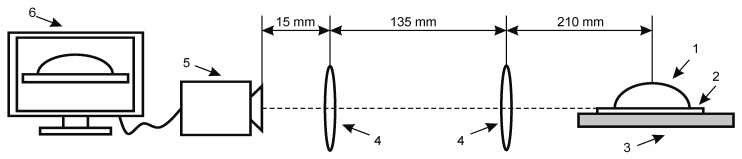
Experimental setup for contact angle measurement of hydrophobic and hydrophilic material consists of (1) drop on specimen, (2) analyzed coating, (3) specimen, (4) double convex lenses, (5) Guppy F–503 B&W CMOS Camera, (6) computer system for analyzing captured image.

Contact angle measurements for wettability and surface free energy evaluation. Knowledge of wetting behavior of solvents on solid surfaces is important for studying intermolecular communication between piezoelectric solid surface and liquid/analyte and to enhance both the mechanical and electrical properties of fabricated samples. The intensity of wetting is identifying the balance of the force between adhesion and cohesion. The experimental set up for contact angle measurement system is presented in Figure 3.

Four different types of liquids were used for measurements of hydrophobicity—water, glycerol, spirit, and olive oil. Main properties are given in Table 2.

Contact angle *θ* is the angle between tangent to a liquid drop at the three phase contact points and solid surface. Thus, critical surface energy of samples was determined from interaction of cohesion and adhesion forces between liquid and solid by observing behavior of a liquid drop on piezoceramics solid surfaces of Ba32, Ba48, ba90, and Ba140 (see Table 3).

Cohesion forces act within a material keeping its molecules together. Work of cohesion is work needed to separate a homogeneous liquid from contact. Cohesion is equal to:

(8) $$W_{11}=2\sigma ;$$

Adhesion forces act between the surfaces of two different materials in contact. It is work, needed to separate two immiscible liquids. It is:

(9) $$\gamma_{12}=\sigma_{1}+\sigma_{2}-W_{12}$$

Surface tension *γ* is determined by cohesion forces acting inside the material. It is determined from force *F* to stretch a liquid film of length *L*:

(10) F=γL

Surface free energy (SFE)—surface energy is a measure of this energy loss. Surface energy per unit area is equal to the half of its energy of cohesion. A free surface is that between water and air or solid and air. Interfacial tension is energy needed to create a unit area of interface.

Contact angle θ_c_ is the angle between tangent to a liquid drop at a three phase contact point and solid surface. Surface energy can be determined from interaction of cohesion and adhesion forces between liquid and solid by observing behavior of a liquid drop on a solid surface.

According to Zisman method [21], SFE of solid is determined using critical surface tension of a liquid. From the previous equation it is determined that interfacial tension *γ_SL_* is equal to the difference of SFE of solid and that of a liquid when contact angle is zero:

(11) $$\gamma_{SL}=\gamma_{SV}-\gamma_{LV}$$

Zisman found that the relationship between cos θ and *σ*_1_ is often linear, so the critical surface tension of wettability is determined empirically using a graph. However, there are few limits of the Zisman method: an interfacial tension is equal to the difference of individual surface tensions and this method does not take into account polar and disperse fractions of surface tension of materials.

**Figure 4:**
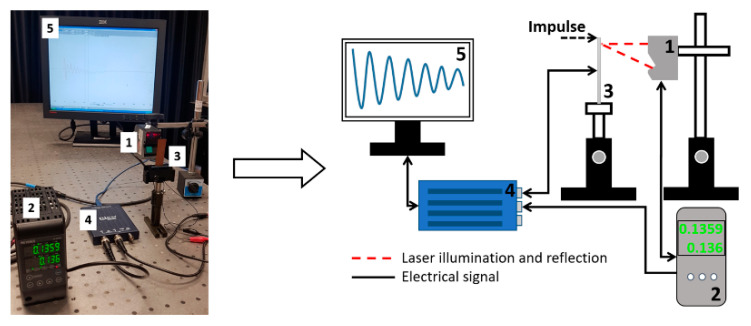
Experimental setup for dynamic and electric characterization of samples: (1) Sensor Head Spot Type Keyence LK–H050, (2) Controller Keyence LK–HD500, (3) sample, (4) PicoScope 2205A, (5) monitor.

Evaluation of piezoelectric properties. For the evaluation of piezoelectric characteristics of the samples, an experimental setup of the high-speed/high-accuracy laser triangulation measurement system (Keyence, Osaka, Japan), together with the USB oscilloscope PicoScope, was used. In this experiment, samples were investigated under direct piezoelectric effect, i.e., a mechanical impulse of 5 N was applied to each sample. This mechanical energy is converted into an electrical energy due to thin film deformation and piezoelectric properties, and then a voltage characteristic is registered and utilized using analogous-to-digital converter (see Figure 4).

ESPI for dynamic characterization. For out of plane vibration measurements an Electronic Speckle Pattern Interferometry (ESPI) based technique (PRISM system) was implemented in the experiment. It is a full-field, noncontact and nondestructive technique meaning that the samples are not destroyed during the measurement and does not require scanning of the surface to observe the vibrations of the sample in different points. Moreover, samples dynamic parameters are not affected by the measurement itself because no additional forces or other disruptions are applied.

**Figure 5:**
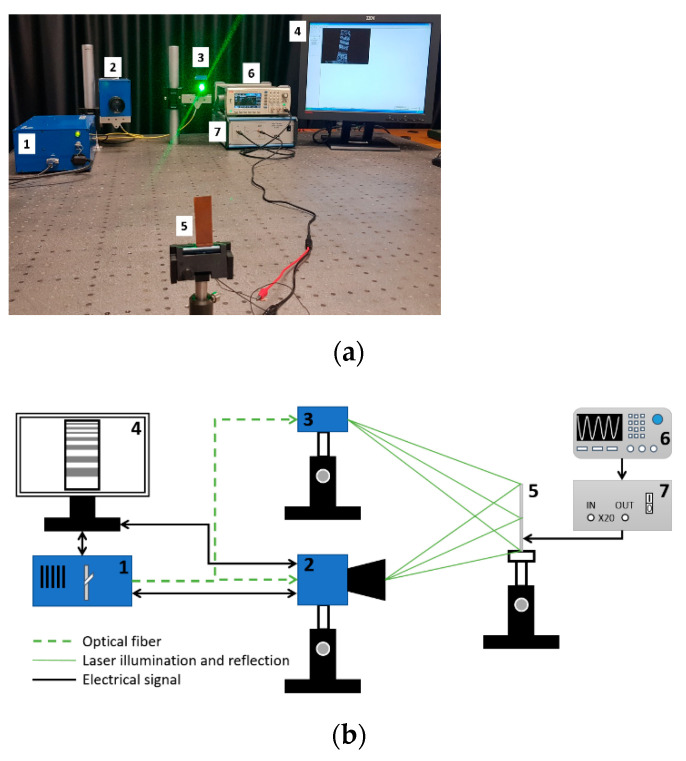
Set-up picture (**a**) and operation scheme (**b**) of the ESPI measurement system: (1) control block, (2) video head, (3) illumination head, (4) monitor, (5) sample, (6) signal generator UNI–T UTG2025A, and (7) high voltage linear amplifier FLC Electronics A400.

In ESPI measurement systems, an investigated sample is illuminated by a laser beam and the image forms due to subjective speckle pattern (Figure 5a). Further, a light beam strikes at point in this speckle image and scatters at a finite area of the object. Parameters like phase, intensity, and amplitude are directly related to the structure of the surface of an object. Thus, a second beam—the reference beam, is superimposed on the camera. When those two light fields interfere (image beam and reference beam), they combine on a video camera where they are recorded. If the object is subject to displacement of deformation, then a new image is subtracted point by point from the first image to get a speckle pattern where black “fringes” appear.

An experimental scheme of a time-average speckle interferometry system for vibrations is shown in Figure 5.

Here (Figure 5b), a laser beam is transmitted by a beam-splitter and directed to the vibrating sample, which is illuminated by this beam. In ESPI, an actuator is implemented for temporal phase stepping. It produces variations of a four-step reference phase. At each step this phase shifts by 0, *π*/2, *π*, and 3*π*/2. These variations (reference phase variations) can be then evaluated using the equation [22]:

(12) $$\Delta \phi_{i}=(i-1)\frac{\pi }{2};$$

where, *i* = 1, 2, 3, 4. Interference wave amplitude $\bar{R}$, occurring between the uniform reference wave *R*, may be expressed as an exponential of it, representing the sampling of the interference field:

(13) $$\bar{R}=R\cdot \exp[j(\phi_{r}-\Delta \phi_{i})];$$

The ESPI reference wave may be either smooth or speckled. A speckled object wave forms due to the plane image of harmonically vibrating sample and camera lens. Thus, a speckled object wave may be evaluated by the exponential formula:

(14) $$\bar{O}=O\cdot \exp[j(\phi_{\nu }\cos\omega t)];$$

During a certain frame acquisition time over a large number of vibration periods, with the help of the camera, an interference field of these waves is integrated and may be described by the formula:

(15) $$I_{i}=m+n\cos[\phi_{o-r}(x,y)+\alpha ]\cdot J_{o}[\phi_{\nu }(x,y)];$$

here, *φ_o–r_*—random phase difference between the uniform reference wave and the speckled object wave corresponding to a point (*x*, *y*) of the object in the equilibrium position. *J_o_*(*φ_ν_*)—the first kind zero-th order Bessel function whose argument is the vibration-related phase. In Equation (15), vibration phase *ϕ_v_* at a point (*x*, *y*) may be expressed through the vibration amplitude *d* at the same point, by formula:

(16) $$\phi_{\nu }(x,y)=\frac{4\pi }{\lambda }d(x,y);$$

here, *d* is the vibration amplitude at the (*x*,*y*) point. Implementation of a four bucket phase mapping algorithm, gives a stabilized system response. Differences of four stepped data in a real-time and post-processing (or two orthogonal data fields) may be expressed by formulas, respectively:

(17) $$C=I_{1}-I_{3}=2OR\cos\varphi_{o-r}\cdot J_{o}(\phi_{\nu });$$

(18) $$S=I_{4}-I_{2}=2OD\sin\phi_{o-r}\cdot J_{o}(\phi_{\nu });$$

Since in the ESPI the sensitivity is very high, all amplitudes are measured simultaneously. Thus, only very small, up to few micrometers, amplitudes are needed to form the fringe pattern on the screen. Therefore, dark fringes (or the minima) on the screen, observed on the vibrating sample (Figure 5), correspond to amplitudes that may be expressed by the formula:

(19) $$d=k\cdot \frac{\lambda }{4};(k=1,2,3\ldots )$$

## 3. Results

In this experimental work, a lead-free piezoelectric ceramic Ba_0.85_Ca_0.15_(Zr_0.1_Ti_0.9_)O_3_ (BCZT) was synthesized. The structure and lattice characteristics, chemical composition, surface morphology, and piezoelectric properties of BCZT based elements (Ba32, Ba48, Ba90, and Ba140) were investigated.

### 3.1. Characterization of BCZT

#### 3.1.1. XRD Used for BCZT Structure and Lattice Characteristics Evaluation

**Figure 6:**
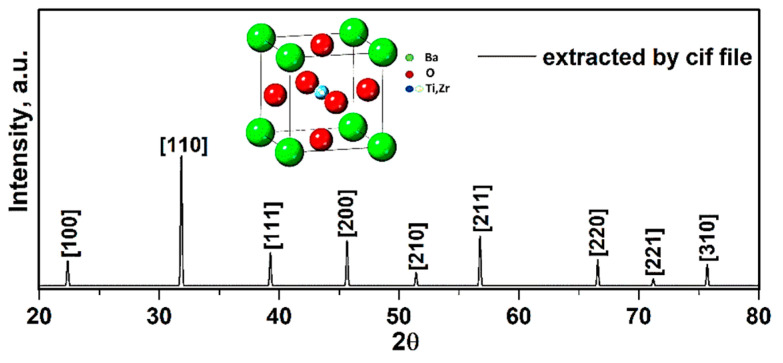
XRD patterns of compound obtained from Cif file.

To study the structure and lattice characteristics of synthesized compound, experimental XRD data was obtained via utilizing the X’Pert software. The X-ray diffraction results of BCZT presence in the chemical solution are presented in Figure 6.

To ensure the formation of the pure structure of compound, the perovskite phase with some impurities was observed. The impurities were determined to be BaCO_3_ and ZrO_2_, in correspondence with the PDF standard cards 44–1487 and 42–1164 (Figure 6). These impurities were confirmed via presence of all elements extracted through the EDX spectra (Figure 9). In addition, these peaks corresponded with the X-ray diffraction data to a standard phase (reference code: 96–433–0785–JCPDS No.05–0626) [23,24]. Moreover, splitting of peaks at 2ϴ~31.35° related to (110) and 2ϴ~45.61° related to (200) plane gives fingerprint of tetragonal crystal symmetry [25]. The calculated values of crystallographic parameters of compound are tabulated in Table 4.

The given XRD pattern (Figure 6) represents the BaTiO_3_ phase, i.e., single perovskite phase with no second phase traces. In previous research, the polymorphic rhombohedra–tetragonal phase transition temperature was found shifted toward room temperature with the Li doping for BCZT [26]. The study showed that BCZT lead-free piezoelectric ceramic with improved performance properties at room temperature can be achieved by shifting the polymorphic phase transition point nearer to room temperature through the addition of LiF [9,26].

The lattice constant *a* (in Table 4) was calculated using Equation (6), i.e., for (110), *a* was 4.03 Å with a crystal structure to be cubic. Additionally, the average BT crystallite diameter D_XRD_ was estimated from the Equation (7), using the data of most intense (110) peak, i.e., the calculated average crystallite size was 0.72 ± 0.05 µm confirming the formation of BT nanoparticles.

#### 3.1.2. Chemical Composition Using FTIR

**Figure 7:**
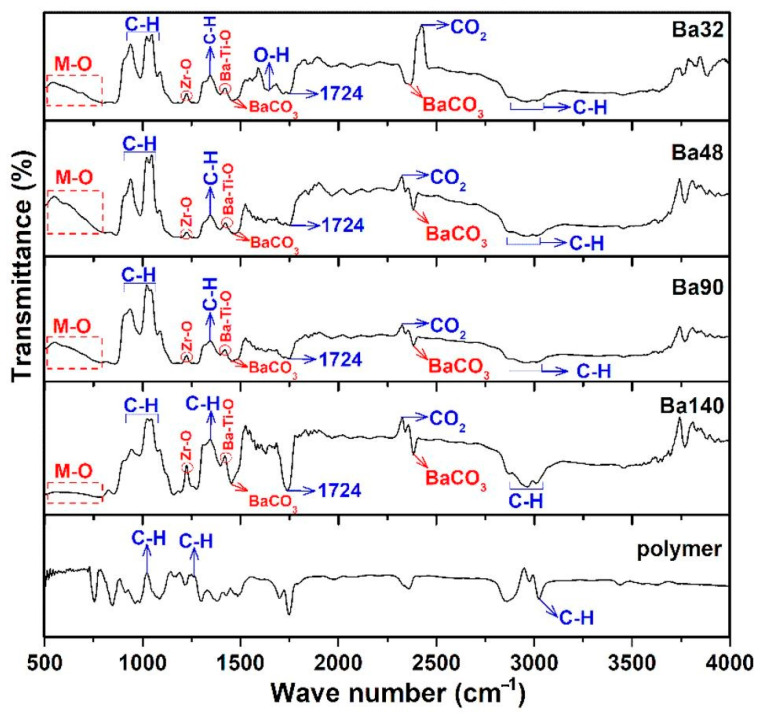
FTIR spectrum of samples Ba32, Ba48, Ba90, and Ba140 showing bending and stretching between molecules in form of peaks in transmittance mode.

Fourier transform infrared spectroscopy (FTIR) analysis of all samples Ba32, Ba48, Ba90, and Ba140 was carried out for investigation presence of functional groups in compounds. Typical FTIR absorbance spectrum was done at 4000–490 cm^−1^ in transmittance mode. According to the Figure 7, several bands associated to the symmetric and asymmetric stretching vibrations of water at the range from 2800 to 3150 cm^−1^ [27], and band of CO_2_ at around 2333 cm^−1^ [28] were observed. Furthermore, the existence of acetate groups bonded to barium atoms and also the asymmetric COO^−^ and symmetric COO^−^ stretching vibrations of carboxylate groups were clearly indicated in the band at 1724 cm^−1^ [29]. The band in the range from 500 to 900 cm^−1^ is related to the bonding of metal with oxygen and it can be attributed to the titanium–oxygen/zirconium-stretching and bending vibrations of the TiO_6_/ZrO_6_ octahedra, respectively [26,30,31]. In addition, the band of Zr–O is existed at ≈1220 cm^−1^ [25]. It is interesting that there are some spectra with very low transmittance ranges, and they are related to the changing nature of materials, chemical reactions, and crystallization of compounds at higher temperatures [32]. The results of the FTIR spectra are consistent with those investigations of XRD and EDX analysis.

Results showed that samples had the same or very similar absorption peaks at the same mode of vibration and shift toward the lower wavenumber and high energy. It may be concluded that thickness of BCZT thin film does not imply the internal bonding of the structures.

### 3.2. Surface Morphology Characterization

#### 3.2.1. SEM and EDX Analysis

**Figure 8:**
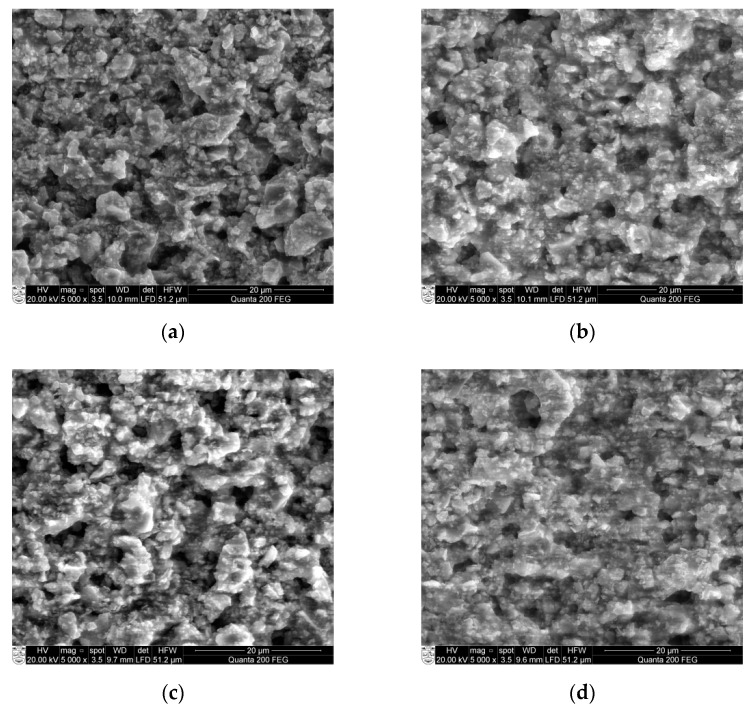
SEM micrographs of: (**a**) Ba32 (**b**) Ba48 (**c**) Ba90, and (**d**) Ba140 samples.

The surface morphology of all four samples Ba32, Ba48, Ba90, and Ba140 was studied by scanning electron microscopy (SEM). The grain size was measured from SEM micrographs too. As seen from Figure 8, BCZT samples have a typical porous structure with small grains of size from 0.2 to 5 µm.

Results in Figure 8 show some pileups and very small crystalline structures formed on the surface. As for example, Ba32 (Figure 8a) has some porosities on the surface with grain size ranging from 0.2 to 7.2 µm. Samples Ba48 and Ba90 have similar surface properties. However, sample Ba140 shows a rather smooth surface with only few pileups with grain size from 0.2 to 2 µm. The distributions of porosities consist of uniformity and mostly they are closed, no open porosities are observed. In this case, the porosity is not a problem in the design of the microchannel because the size of porosities is less than 100 micron, therefore, they cannot damage or influence the mechanical parameters directly.

**Figure 9:**
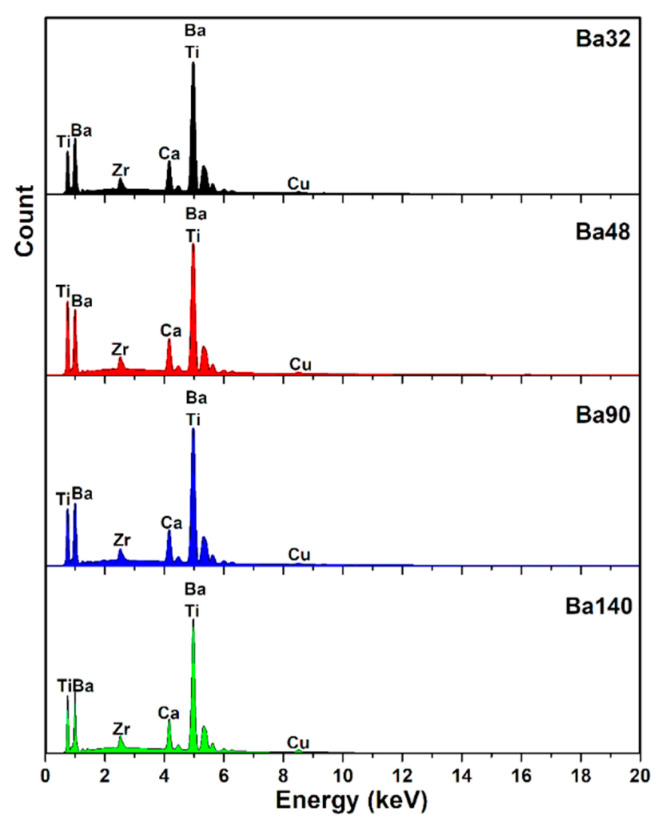
Energy dispersive X-ray analysis (EDX) spectra of compounds.

Taking into account the compositional distribution of compounds, the energy dispersive X-ray (EDX) spectrum is shown in Figure 9.

In addition, the weight presence of elements such as C, O, Ca, Ti, Zr, Br, and Cu (as the background) is listed in Table 5. EDX analysis of compounds was in good agreement with results of X-ray diffraction and FTIR.

#### 3.2.2. Wettability

For investigations of samples surface wettability, a Zisman [21] method was used to determine the critical surface tension after measuring the contact angle *θ*.

**Figure 10:**
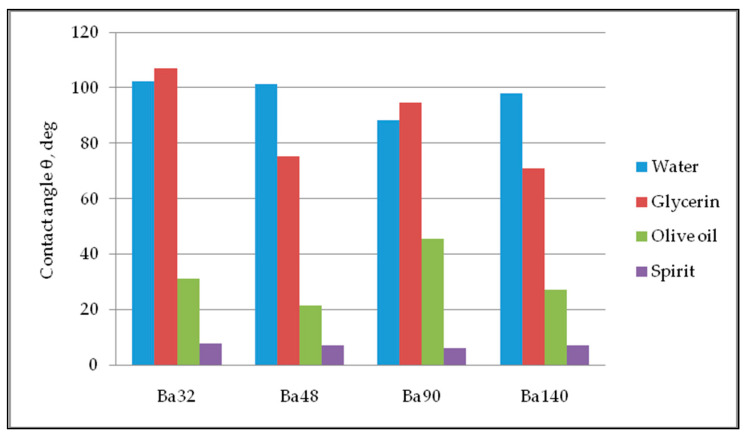
Diagram of contact angle measurements.

Five repetitions of contact angle measurements of each sample were done for precise calculations with an obtained error bar of 0.31–0.98 degrees. The values of average contact angle measurement are registered in Figure 10. Thus, all samples have partial wetting hydrophilic surface, with high degree of wettability and strong solid/liquid interactions for liquids glycerin, olive oil, and spirit. For water, all samples showed hydrophobic properties with low wettability and week solid/liquid interaction.

In a Zisman investigation, the cosine of the contact angle θ is plotted versus the surface tension of the appropriate liquid. The value of the surface tension extrapolated to cos(θ) = 1 (contact angle = 0°) is referred to as the critical surface tension *σ_crit_*.

**Figure 11:**
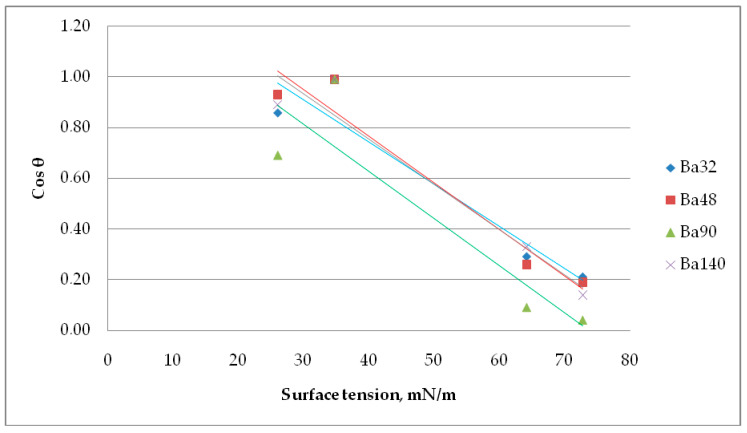
Determination of the critical surface tension using a Zisman method.

Results from Figure 11 imply that the critical surface tension for Ba32 is *σ_crit_* = 24.69 mN/m, for Ba48 *σ_crit_* = 27.20 mN/m, for Ba90 *σ_crit_* = 20.05 mN/m, and for Ba140 *σ_crit_* = 26.38 mN/m. This value is often named as the surface free energy of solid. The contact angle of liquids is dependent on the material and its chemical nature. The number and type of sites at the interphase, the surface texture, and surface roughness, all have direct effects on the surface wetting of piezoceramics.

In this case, homogeneous nucleation occurs rarely. Virtually, it is always the case that nucleation processes are catalyzed by heterogeneity, such as an accommodation of a substrate surface. The different ambient is viewed as an example of **heterogeneous nucleation**. Due to the bonding across the substrate interface, less energy is expended in creating the cap heterogeneously, than in creating a spherical nucleus homogeneously. In this case, nuclei that solidify onto a mold wall are mostly similar. Due to the effective reduction in surface energy, the critical nucleus is smaller and the rate of formation is greater relative to that obtained by homogeneous nucleation. Heterogeneous nucleation is difficult to analyze not only because of the geometry, but also because of the often unknown interactions between the nucleus and substrate.

### 3.3. Piezoelectricity in Lead-Free BCZT Composites

**Figure 12:**
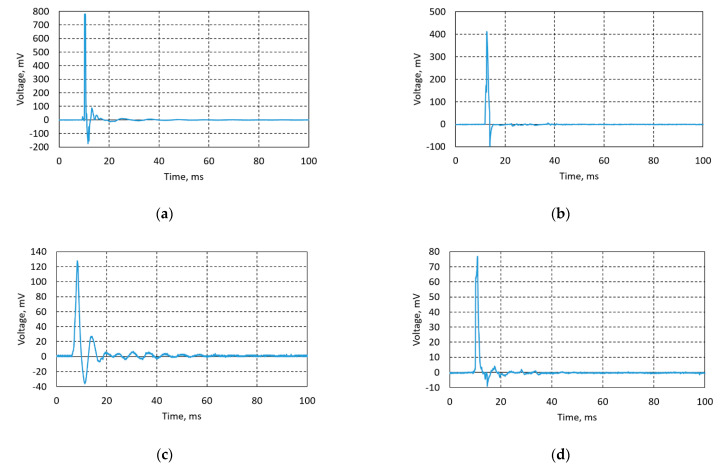
Generated voltage diagrams of (**a**) Ba32, (**b**) Ba48, (**c**) Ba90, and (**d**) Ba140.

Lead-free piezoceramic samples Ba32, Ba48, Ba90, and Ba140 were optimized for their synthesis and performance conditions in order to maximize the piezoelectricity effect. Experiments were done using direct piezoelectric effect, i.e., an impulse constant force of 5 N was applied on the samples and a voltage characteristic was registered using the data acquisition system USB oscilloscope PicoScope. Voltage was measured in an open circuit. Thus, designed BCZT based elements generated from 78 mV up to 782 mV (Figure 12).

Results, given in Figure 12a, state that sample Ba32 generates the highest amount of energy—782 mV. Sample Ba48 (Figure 12b) generated 410 mV, sample Ba90 (Figure 12c) generated 130 mV, and sample Ba140 (Figure 12d) generated 76 mV. Obtained experimental data showed significant results of lead-free piezoceramics comparing it with previous research related to the PZT ceramics [13], i.e., the difference of generated voltage is obvious compared to PZT based piezoceramics investigated in previous research, where PZT based piezoceramics generated from ≈1.4 to ≈3.5 mV [12,13,20]. The difference in results is also related to thickness of the samples, i.e., when thickness decreases its stiffness decreases too. Therefore, natural frequency is lower, and the peaks observed in Figure 12 are wider.

An important parameter in investigation of piezoelectric properties of BCZT based elements is related to its Young’s modulus of elasticity. It determines the conversion of strain to force which was delivered by the actuator. Therefore, experimental results showed that Young’s modulus of the samples is very low, only 0.8–1.25 GPa. This value is very similar to PMMA Young’s modulus of elasticity—1.8–3.1 GPa [33]. In comparison to pure BCZT, its Young’s modulus is around 118 GPa [34], so about 10 times bigger. However, obtained results are in accordance with the literature, which states that if material has lower Young’s modulus, then it will have much higher piezoelectric coefficients [34]. Moreover, piezoelectric material with higher thickness has much higher d33 piezoelectric coefficient, therefore, the generated voltage is higher too [35].

Several studies have shown that a fine domain structure of barium titanate (BT) material during the synthesis routes can greatly improve its dielectric and piezoelectric properties. For BT at room temperature, d_33_ values ranging from 350 [36] to 500 pC/N and d_31_ = −185 pC/N were achieved [34]. Thus, PZT d_33_ piezoelectric coefficient varies from 152 to 593 pC/N [37]. When PZT is mixed with polymeric materials, its d_33_ piezoelectric coefficient decreases. Depending on the concentration and polymer type, d_33_ ranges from 48 to 50 pC/N [38,39]. In this research, BCZT was synthesized with PMMA polymer which led to d_33_ piezoelectric coefficient of 46 pC/N, with BCZT volume fraction number 0.8 (see Table 6).

Moreover, considering the grain size of ferroelectric thin films or electrodes, the influence of grain size and grain boundary cannot be neglected, and ferroelectric films and electrodes are required to be thin on the piezoelectricity, but in this case, there is an optimum value of thickness. For this reason, it is necessary to investigate the influence of grain size, grain boundary, and thickness on the electrical properties. The size effects of thin films are different from that of bulk materials. Size effects of thin films include not only grain size but also film thickness. It is difficult to distinguish the size effects derived from grain size from those derived from film thickness, because the grain size of the thin films generally changes with film thickness. Furthermore, it has also been reported that the film thickness dependence of a polycrystalline, such as in this study, film is different from that of epitaxially grown films. In this case, composite has a tetragonal structure over the entire thickness range investigated. The composites possess a dense and granular microstructure, whereas the thicker sandwich has grain, aggregation related to the rigidity in Cu substrate, and randomly oriented perovskite phase. The thinner sandwich panel tends to increase the leakage current as well as decrease the dielectric constant and piezoelectric coefficient due to the internal strain resulting from the interfacial layer. The optimal thickness is very important, and the results of this study are very promising and suggesting that this sandwich panel can be used as a storage element in nonvolatile electric random access composite.

### 3.4. BCZT Based Element for Acoustic Particle Manipulation

#### 3.4.1. Dynamic Properties

Designed lead-free BCZT piezoceramics demonstrate good piezoelectric properties at micrometric level. Therefore, it could be used for selection, sorting, and manipulation of particles in various fluids using ultrahigh frequency excitation or acoustophoresis.

**Figure 13:**
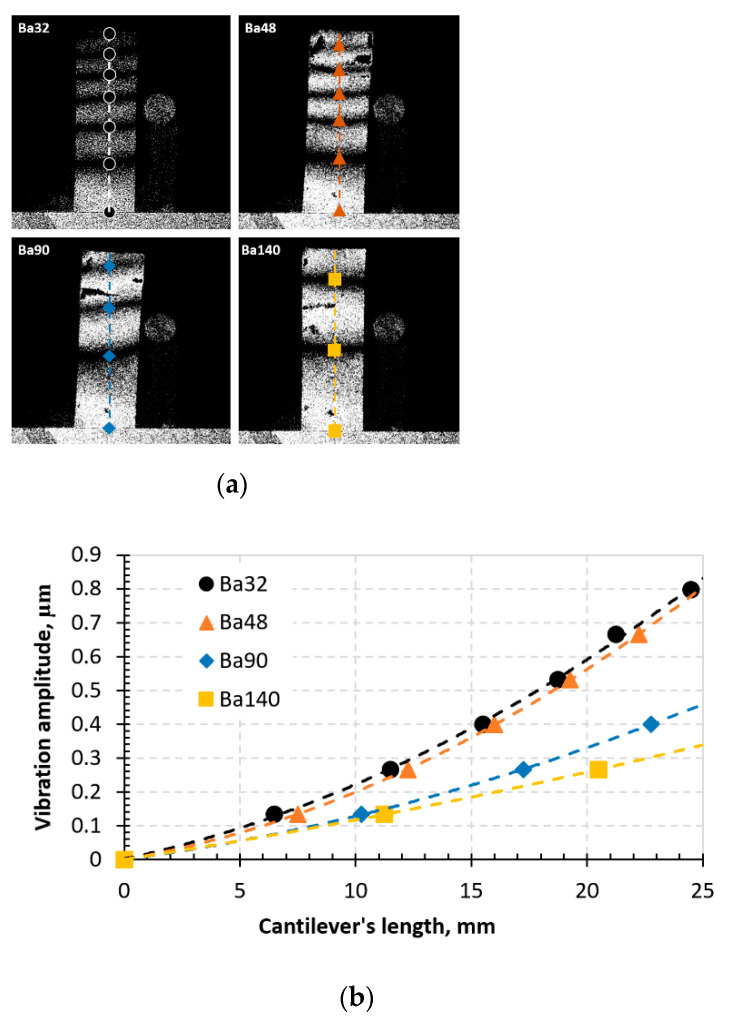
Time-average speckle interferogram (**a**) and graph representing vibrational modes in the BCZT elements (**b**).

During the experimental procedure, the typical aspects of time-averaged fringes were observed on the monitor while the samples were vibrated. The images are given in Figure 13.

From the time-average speckle interferogram (Figure 13a), dark fringes indicate the vibrations of the designed elements in both ends in horizontal sense. Results showed that an indirect piezoelectric effect is observed in all samples, however, element Ba32 shows the highest piezoelectricity compared to others. The lowest indirect piezoelectric effect is observed in element Ba140. This indicates that Ba32 has the highest sensitivity to periodical electrical excitation.

#### 3.4.2. Design of Microfluidic for Particle Manipulation Using Bulk Acoustic Waves

**Figure 14:**
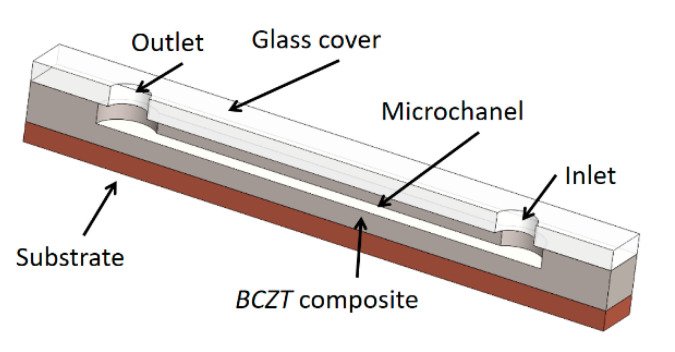
Prototype of microfluidic device with implemented BCZT composite.

Implementation of designed BCZT based element for acoustic particle manipulation is based on the bulk acoustic waves (BAW). Designed element excites bulk waves and resonance in channels through the material (here a BCZT based element). Potential application is based on a droplet processing microfluidic systems given in Figure 14.

The general scheme of a designed BCZT based microfluidic device is composed of a substrate, on the top of the substrate a lead-free BCZT composite layer is formed together with the microchannel in it (Figure 14). A glass cover with the inlet and outlet holes covers up the microchannel. The width of the microchannel is 350 µm, height is 100 µm, and its length is 20 mm, respectively. Thus, a microchannel was formed using thermal imprint assisted by high frequency vibrations [40].

**Figure 15:**
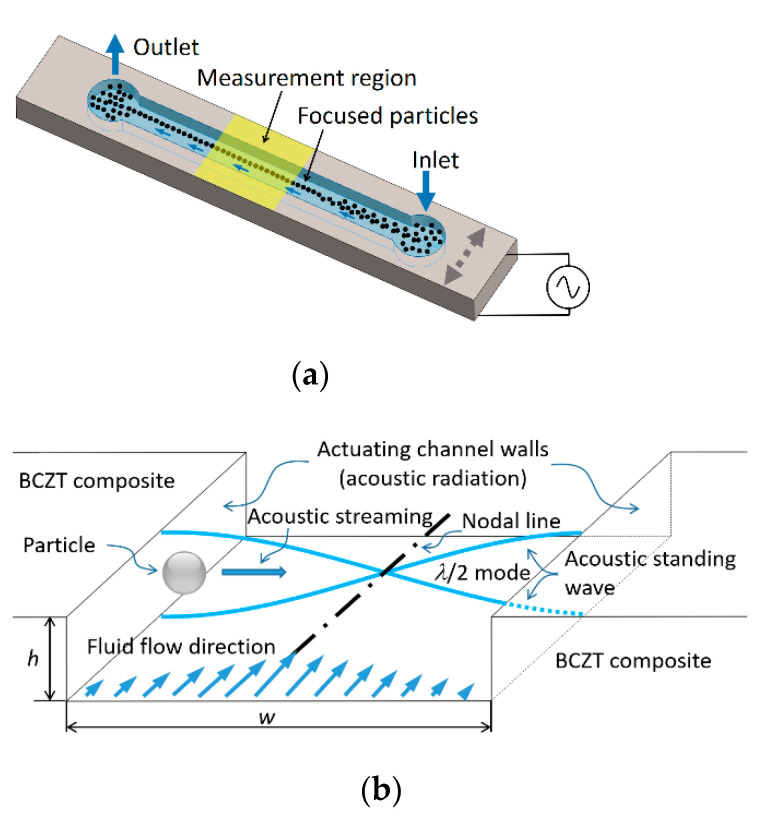
3D view of the microchannel device (**a**) and its working principle (**b**).

An operating principle of designed microfluidic system based on acoustophoresis is given in Figure 15.

A designed microchannel (Figure 15) consists of the inlet and outlet holes through which the fluid is passed. The particles can be manipulated using BAW acoustophoresis effect when the fluid continuously flows through the inlet. In Figure 15a, the region “measurement region” is the area where particles are focused on the channel centerline. When a channel is tuned using BAW (Figure 15b) due to actuating channel walls, an acoustic radiation appears. A resonance mode of *λ*/2 standing pressure wave is formed. In this case, the resonance frequency can be defined by equation:

(20) $$f=\frac{c}{\lambda };$$

here, *c*—speed of sound in liquid; *λ*—wavelength. Speed of sound in fluids can be evaluated by equation:

(21) $$c=\sqrt{\frac{K}{\rho }}$$

where *K*—bulk modulus of elasticity (N/m^2^); *ρ*—density (kg/m^3^). Bulk modulus of elasticity of water is *K* = 2.15 × 10^9^ N/m^2^, density *ρ* = 999.8 kg/m^3^. Thus, calculated speed of sound in water at 20 °C temperature is c = 1466.4 m/s. Wavelength *λ* is directly related to the width of microchannel, i.e., for half wave standing pressure *λ*/2 = *w*. In this case, width of microchannel is *w* = 350 µm. Thus, the resonance frequency, required to focus the particles in the middle of microchannel (as shown in Figure 15a measurement region) is *f* = 2.1 MHz.

In order to evaluate the dynamics of fluid, it is necessary to evaluate Reynolds number. It shows whether the flow is turbulent or laminar. In the case of turbulent flow, the particles move irregularly, and the Reynolds number is more than 4000. In the case of laminar flow, Reynolds number is less than 2000 and particle motion is regular and smooth. However, due to downscaling, Reynolds number in microfluidic channels can be evaluated using equation:

(22) Re=ρuDHμ

where *ρ* is the fluid density; *u*—fluid flow velocity (m/s); *D_H_* is a hydraulic diameter of microchannel (m); *µ*—dynamic viscosity (Ns/m^2^). Fluid flow rate is defined as *v* = 5 µL/min. Fluid flow speed *u* could be calculated by equation $u=\frac{v}{A}$, where *A*—microchannels cross-sectional area (m^2^). Therefore, *u* = 0.00238 m/s. Water density at 20 °C is *ρ* = 999.8 kg/m^3^, and its dynamic viscosity *µ* = 0.0010005 Ns/m^2^. A hydraulic diameter is determined from the equation $D_{H}=\frac{4A}{P}$, where *A*—microchannels cross-sectional area (m^2^); *P*—perimeter of the microchannel. Thus, a hydraulic diameter is $D_{H}=156\times 10^{-6}$ m. Therefore, according to Equation (22), experimental value of Reynolds number is 0.37, defining that the flow is laminar and viscous forces dominate over inertial.

#### 3.4.3. Experimental Investigation of Particle Motion in Microchannel under Effect of BAW

**Figure 16:**
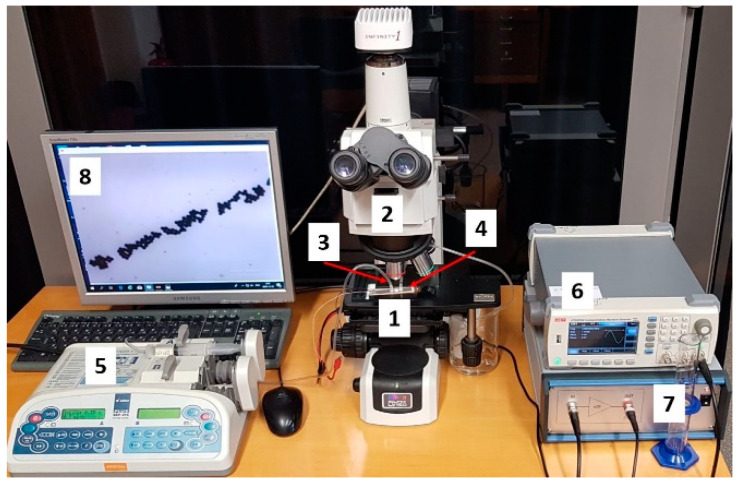
Microfluidic testing system consisting of (1) microfluidic, (2) Microscope Nikon ECLIPSE LV150, (3) inlet connection, (4) outlet connection, (5) syringe pump Aitecs PLUS SEP–21S, (6) signal generator UNI–T UTG2025A, (7) high voltage linear amplifier FLC Electronics A400, and (8) monitor.

For experimental investigation of particles control using bulk acoustic waves, a microfluidic channel was connected to syringe pump (Aitecs PLUS SEP–21S, Viltechmeda, Vilnius, Lithuania) via inlet connection. Here, fluid actuation through the microchannel was achieved by pressure driven flow, where the fluid was pumped through the microchannel with the help of positive displacement pumps (Figure 16). Used fluid from outlet connection was then directed to the discharged vessel. A signal generator (UNI–T UTG2025A, Uni-Trend Technology, Dongguan, China) and a high voltage linear amplifier (FLC Electronics A400, Pendulum Instruments, Drottningskar, Sweden) were used for generation of bulk acoustic waves. The flow of particles in microchannel was observed with microscope (Nikon ECLIPSE LV150) and registered with Infinity program.

**Figure 17:**
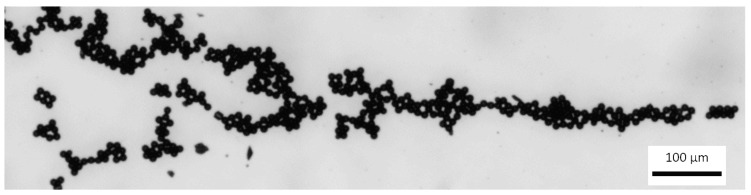
Focused particles in the microchannel (scale size 100 µm).

Based on the laws of fluid mechanics when a pressure driven laminar flow is observed in microchannel, the fluid velocity at the walls must be 0 (a no-slip boundary condition). During the flow process, in a microchannel, bulk acoustic wave condition creates a parabolic velocity profile (Figure 17) with focused polystyrene particles of 10 µm diameter. Flow rate was 5 µL/min. According to the Figure 6, we encountered crystalline (polycrystalline) grains and there were no amorphous or mixed structures observed. Clearly, crystallization can help in creation of separation particles. In addition, visualization of the presented design provides a small and well-characterized bulk acoustic wave device by fabricating separation particles, i.e., separation particles can be created by a filter having a wide band width or a resonator having a wide oscillation frequency range together with a circuit.

Experimental data showed that using frequency of 1.89 MHz, polystyrene particles were focused in the central line for bulk acoustic wave generation. Obtained frequency is about 11% smaller compared to the theoretical.

## 4. Novelty and Discussion

Previous research was concentrated on design of PZT based piezoceramics [12,13,20] for MEMS and NEMS purposes. This research was concentrated on creating lead-free piezoceramics which could be implemented in designing an effective manipulation microdevice able to control a large variety of biomolecules or particles in different suspending mediums based on acoustophoresis or BAW.

A bulk acoustic wave method allows designing various configurations of devices and channel geometries. Devices based on BAW typically work at a lower frequency range of about 1.0–10 MHz. Lower operation frequencies condition longer wavelengths, enabling to handle droplets up to 500 µm in the channel up to 1 mm width. Moreover, using BAW it is possible to scale down device size and the frequency in order to accommodate smaller droplets. Therefore, the basic advantage of the presented BCZT composite material is its effective generation of bulk acoustic waves, low operation frequency, biocompatibility, and its thermal stability. Usually, for formation of BAW in microfluidic channels, piezoceramic PZT is used. However, there are many observed drawbacks affecting particle migration in the flow when PZT is implemented, for example, heating, which can damage biological particles in the flow (as proteins). Misfolding and aggregation of bioparticles is caused by this thermal stress, a rapid increase in temperature [41]. Therefore, the usage of PZT ceramics in the design of such microfluidic channels is crucial for thermally sensitive proteins. Another drawback is compatibility of lead with bioparticles. Thus, lead-free BCZT composite solves all these drawbacks because it generates BAW, has good thermal stability, and is a lead-free biologically compatible material.

For future research, implementation of silver nanoparticles in BCZT matrix is foreseen. It would allow BCZT composite material to be used in medicine for bioparticle identification using laser technologies because of surface plasmon resonance (SPR) effect.

The designed lead-free BCZT based microfluidic device for controlled particle flow manipulation based on BAW offers significant benefits for fluid handling instruments in pharmaceutical and biochemical research, laboratory diagnostics, etc.

## 5. Conclusions

Designed lead-free BCZT composite material was successfully investigated and implemented in the design of BCZT based elements used for microchannels for acoustic droplet handling systems.

Experimental results of FTIR and XRD confirmed the formation of BCZT. From XRD data, the calculated average crystallite size was 0.72 ± 0.05 µm confirming the formation of BT nanoparticles. FTIR results showed that BCZT thin film thickness does not influence chemical composition, i.e., all four samples had the same or very similar absorption peaks at the same mode of vibration and shift toward the lower wavenumber and high energy, leading to the conclusion that thickness of BCZT thin film does not imply the internal bonding of the structures.

Thus, SEM micrographs showed that thin films formed from synthesized BCZT have few small cavities on surface with grain size ranging from 0.2 to 7.2 µm.

Measurements of wettability showed that samples have a partial wetting hydrophilic surface, with high degree of wettability and strong solid/liquid interactions for liquids. Calculated critical surface tension for all samples was very similar, ranging from 20.05 to 27.20 mN/m.

Direct piezoelectric test showed significant results of lead-free piezoceramics, i.e., under constant 5 N mechanical impacts thin films generated from 76 mV up to 782 mV voltages. Moreover, experimental results showed that Young’s modulus of the samples is very low, only 0.8–1.25 GPa leading to much higher piezoelectric coefficients compared to other materials.

Experimental data was used in numerical simulation for design of a microchannel numerical model for the acoustic droplet handling system to show the manipulation and sorting of particles in fluid using bulk acoustic waves. In this case, BCZT based element had a Reynolds number of 0.37, meaning that the flow is completely laminar, and no turbulence occurs in fluid flow in the microchannel. Experimental data showed that using frequency of 1.89 MHz, polystyrene particles were focused in the central line for bulk acoustic wave generation. The obtained frequency was about 11% smaller compared to the theoretical one.

Integration of lead-free BCZT piezoceramics thin films in design of microchannels may create multidimensional bulk acoustic waves used to control the migration and concentration of particle kinetics. This feature is desirable in such microsystem devices where scalability, programmability, and the ability to control variable size particles is crucial.

## Figures and Tables

**Table 1:** Samples fabricated during experiments.

Name	Screen Printing Mesh	Mesh Count	Thread Diameter	Open Area	Mesh Thickness	Theoretical Ink Volume	Measured Thickness
		n/cm	µm	%	µm	cm^3^/m^2^	µm
Ba32	32/83–70 PW	32 ± 1.2	70	59	116 ± 6	68	109 ± 5
Ba48	48/123–70 PW	48 ± 1.2	70	41	114 ± 6	47	102 ± 5
Ba90	90/230–48 PW	90 ± 2.5	48	25	76 ± 4	19	86 ± 5
Ba140	140/355–34 PW	140 ± 3.5	34	19	52 ± 3	10	70 ± 5

**Table 2:** Liquid properties.

No.	Liquid	Surface Tension	Density
		mN/m	kg/m^3^
1	Water	72.8	997
2	Glycerol	64.2	1260
3	Spirit	26.02	793
4	Olive oil	34.76	888.89

**Table 3:** Droplet images of different liquids on lead-free piezoceramic samples.

No.	Liquid	Ba32	Ba48	Ba90	Ba140
1.	Water	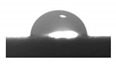	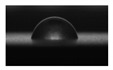	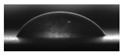	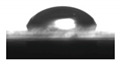
2.	Glycerin	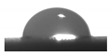	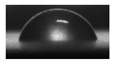	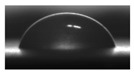	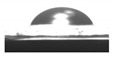
3.	Olive oil	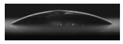	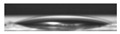	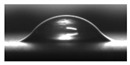	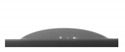
4.	Spirit	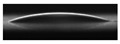	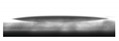	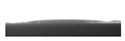	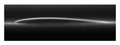

**Table 4:** Crystallographic parameters related to the compound structure obtained through the X’Pert software.

Crystal System	a	c	Cell Volume	Crystal Density	Space Group
	Å^3^	Å	Å^3^	g/cm^3^	
Tetragonal	4.03	4.04	65.68	5.83	P4/mm

**Table 5:** Stoichiometric composition of compounds.

Element	Weight, %			
	Ba32	Ba48	Ba90	Ba140
Carbon	13.10	18.81	15.13	16.03
Oxygen	38.87	40.12	39.54	39.84
Calcium	3.89	3.60	3.74	3.60
Titanium	19.46	16.21	18.64	18.23
Zirconium	2.45	2.20	2.16	2.34
Barium	21.47	18.38	19.84	18.57
Copper	0.72	0.63	0.93	1.35
Sum	100	100	100	100

**Table 6:** Piezoelectric properties of various types of piezoelectric composites.

Type	d_33_
	pC/N
BT [36]	350
SG–BCZT [34]	500
PZT–2 [37]	152
PZT–5H [37]	593
PVDF/0.67 PZT [38]	48
Epoxy/0.685 PZT [39]	50
BCZT/PMMA	46

## Data Availability

Data sharing not applicable.
